# Combining simulations and experiments – a perspective on maximum entropy methods

**DOI:** 10.1039/d5cp01263e

**Published:** 2025-07-02

**Authors:** Johannes Stöckelmaier, Chris Oostenbrink

**Affiliations:** a Institute of Molecular Modeling and Simulation (MMS), BOKU University Vienna Austria chris.oostenbrink@boku.ac.at; b Christian Doppler Laboratory Molecular Informatics in the Biosciences, BOKU University Vienna Austria

## Abstract

To elucidate the connection between the structure and function of intrinsically disordered proteins (IDPs) a description of their conformational ensembles is crucial. These are typically characterized by an extremely large number of similarly low energy conformations, which can hardly be captured by either experimental or computational means only. Rather, the combination of data from both simulation studies and experimental research offers a way towards a more complete understanding of these proteins. Over the last decade, a number of methods have been developed to integrate experimental data and simulations into one model to describe the conformational diversity. While many of these methods have been successfully applied, they often remain black-boxes for the scientist applying them. In this work, we review maximum entropy methods to optimize conformational ensembles of proteins. From a didactical perspective, we aim to present the mathematical concepts and the optimization processes in a common framework, to increase the understanding of these methods.

## Abstract 2

To elucidate the structure of intrinsically disordered proteins (IDPs) a refined description of their conformational ensembles is crucial.

## Graphical abstract

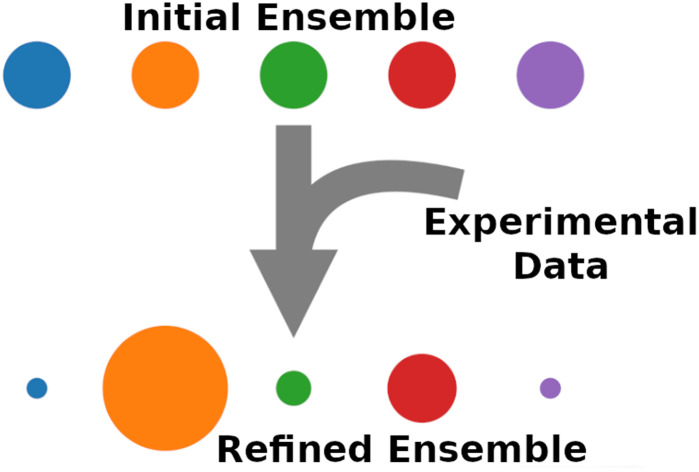

## 1 Introduction

The reproducible folding of biopolymers into functional enzymes, receptors or structural entities is described as the foundation of structural biology and one of the key enablers of life. The observed correlation between amino acid sequence and geometric structure led to the theory of structure–function relationship and has been a solid pillar in the understanding of biochemistry^1,2^ since the mid-20th century. New scientific insights started to weaken this dogma in the early 2000s, revealing that proteins can be classified into different levels of overall structural stability.^3^ Structured proteins feature a well-defined 3D geometry that is thermodynamically stable, while increased flexibility is observed with disordered proteins. Proteins lacking a stable geometry entirely are named intrinsically disordered proteins (IDPs^4^), those that are partly disordered are said to contain intrinsically disordered regions (IDRs).^5^

**Fig. 1:**
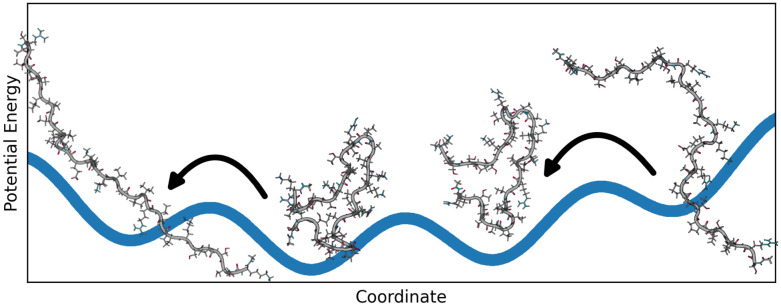
Flatter parts of the potential energy landscape may span multiple conformations. This allows flexible proteins to switch between conformations rapidly at room temperature. The visualization in this figure shows such example where a polypeptide can compact and expand quickly due to its flat potential energy surface. All accessible structures then make up the conformational ensemble, a model to describe flexible proteins.

The elucidation and characterization of structures and the associated dynamics of flexible proteins turned out to be a substantial scientific challenge that requires a close cooperation between experimental studies, data science and molecular simulations.^6–8^ Flexible proteins are often characterized by complex, multifunneled potential energy landscapes with multiple, often shallow, minima.^9,10^ Flatter parts of the landscapes may span multiple conformations, allowing rapid switches between them at ambient temperatures^11^ as visualized in Fig. 1. The observable molecular properties cannot be fully explained by a single structure and therefore it is necessary to create an appropriate representation of the structural diversity. A frequently used model consists of a superposition of different geometric structures, each showing a single relevant structure. The observable molecular properties then emerge as an average over the different structures. All of those structures together represent the conformational ensemble^12–14^ which is a set of molecule geometries with an affiliated probability coefficient or weight.^15^ The true amount of conformations in an ensemble is unknown and depends on the definition of discrete conformations, but can grow very large even with mid-sized molecules.^16,17^

Many established computational methods like comparative modeling^18^ and AI-based structure predictors like Rosettafold^19^ or Alphafold^20^ are designed to calculate static structures of stable proteins. The extension of these methods to also describe conformational ensembles, which are typically described by the sampling of the relevant conformations, is currently a major topic of research.^21–24^ Alternatively, molecular dynamics (MD) simulation uses the ergodic theory, which predicts that a conformational ensemble is captured by following the molecular motions of a molecule over a sufficiently long time. The computational challenge of appropriately sampling all conformations is closely related to the MD simulation of protein folding^25–27^ which has, while still being very challenging especially for larger proteins, seen substantial improvements of parameters and methodology. MD simulation can be applied to investigate the dynamic nature of an IDP and to generate an ensemble. The ensemble obtained with such method contains both conformations and associated probability coefficients. For a straightforward MD simulation, which follows the appropriate equations of motion based on an accurate energy function, and from which conformations are sampled at regular time intervals, the probability coefficients would be identical for all samples. The ensemble can subsequently be reduced in size to group very similar structures into single conformations and to assign their weights according to the occurrence of these conformations in the larger ensemble.

The complex potential energy surfaces of most IDPs and flexible proteins make these probability coefficients prone to errors due to force-field inaccuracies. To obtain not just valid geometrical structures but also the correct associated weights, it is necessary to model not just the well populated conformational minima but also to describe the (reversible) transitions from one conformation to the next and the associated energy barrier correctly.^28^ If the transitions between conformations are not observed sufficiently often, the weights assigned to specific conformations belonging to different minima may not be statistically robust. To address this challenge and to refine the weights of the geometrical ground states it thus seems reasonable to optimize weights *a posteriori* after completing the simulation. A fundamental prerequisite for the successful reweighting of ensembles lies in the complete sampling of the conformational space, often necessitating enhanced sampling methods. Reweighting methods depend on a reasonable sampled conformational space as they cannot create new conformations by them self, but are designed to create an appropriate ensemble from an existing set of conformations to better reproduce experimental data. Thus, initial ensembles obtained from such enhanced sampling methods featuring a wide set of relevant conformations with lower confidence statistical weights represent an ideal use case for *a posteriority* reweighting.

In the last decade, numerous methods have been developed to correct and improve computationally obtained ensembles by optimizing the associated weights using experimental data. Since then, these reweighting methods became an established tool in computational structure elucidation of flexible proteins.^29–32^ The aim of this study is to review some of the most prominent reweighting techniques and to give insights into what are often considered black box methods.

## 2 Refinement of ensembles

As described in the introduction, MD simulations are used to study the behavior of large chemical and biophysical systems, enabling the calculation of *in silico* estimates of the system's biophysical properties. Applying the physical laws of motion, computers can approximate the movement and dynamics of the biophysical system. The forces on atoms, used in the equations of motion, are typically derived from a force field, as an approximation of the interactions between atoms and molecules, more precisely described by quantum mechanical principles. The physical ensemble of the system of interest is obtained from the trajectory in time, sampled at *N* regular intervals, with each conformer having a weight of 1/*N*. *In silico* estimates of observables are initially calculated individually from single conformers of the trajectory and may later be averaged with estimates from other conformers. Therefore, the choice of conformers to calculate an observable is of high importance as different geometrical structures may yield slightly different values for an observable as many are sensitive to conformation.

Experimental observables may also give insight into the relevant conformations of a biomolecule. Particularly insightful for IDPs is nuclear magnetic resonance (NMR) spectroscopy, offering *e.g.* chemical shifts, ^3^*J*-coupling constants, residual dipolar couplings (RDCs) and paramagnetic relaxation enhancement (PRE). During an experimental measurement, a very large number of molecules is measured simultaneously and the averaging timescales are typically long with respect to the molecular motion. Consequently, the measured observables represent directly both a time and ensemble average of the measured molecules.^33–36^ It is therefore invalid to compare observables calculated from a single conformation to the ensemble-averaged experimental results. Accordingly, it is necessary to compute the expectation value for each observable from a representative set of conformations (*i.e.* the computationally derived ensemble) to accurately compare results of experiment and simulation. In many cases, a weighted average over the simulation trajectory is calculated. Eqn (1) shows such an averaging where the ensemble average 〈*O*^calc^〉, indicted by angular brackets, is calculated. The ensemble consists of in total *N* conformations and each conformer *t* has an individual calculated observable *O*^calc^_*t*_ and a statistical weight *w*_*t*_:1
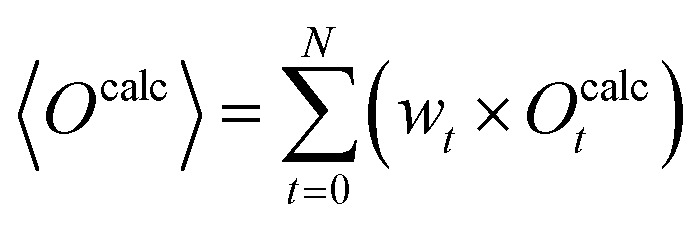


This approach is valid for most experimental data, but not for residual dipolar couplings (RDCs) and nuclear Overhauser effects (NOEs), where different averaging schemes are required. Before the calculation of ensemble averages, the physical nature of each type of observable needs to be considered and the correct averaging scheme must be chosen. For example, NOEs arise from dipolar coupling between the nuclear spin of two protons. The intensity of such signals is highly dependent on the distance in space between a given proton pair and weakens proportional to the third or sixth power of the distance, depending on the timescale of the experiment and the tumbling time of the molecule.^37^ Pairs closer than 3 Å result in strong NOE signals while the limit of detection is reached with pairs 6 Å apart. In ensemble averaging, this means that a small number of conformations with short distances between a proton pair have a dominating influence on the intensity of the NOE signal. To reproduce this behavior, NOE-derived distances require *r*^−3^ or *r*^−6^ averaging,^38,39^*e.g.*:2
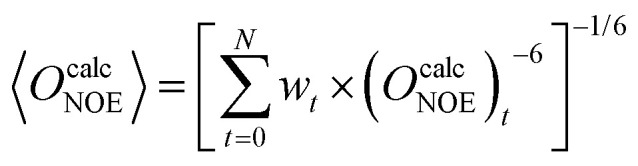


Interpreting the weights, *w*_*t*_, in eqn (1) and (2) as probabilities that the conformation *t* occurs in the ensemble, leads to the condition that the sum of all individual probabilities needs to be one:3
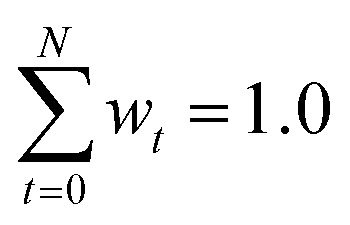


Due to the approximate nature of force fields it is unavoidable to introduce some level of inaccuracy into the simulation. In some simulation settings, such as those involving intrinsically disordered proteins (IDPs), these small inaccuracies are of increased relevance, as most force fields are originally optimized for stable proteins, and can potentially affect the prediction of the observables of the system. The simulated and ensemble-averaged observables, as obtained from the conformational ensemble, may be compared with those measured in experimental studies to confirm the validity of the simulations, identify differences and possibly to correct the simulation to allow further investigation into the properties of the system.

To validate and optimize molecular ensembles, a set of techniques known as reweighting methods can be applied. The basic principle of all of these methods is similar: an initial probability density representing the weights of each conformation of the unbiased ensemble is transformed into a probability density which represents the refined ensemble, aiming to improve the agreement between computationally and experimentally derived ensemble averages of the biophysical observables.

**Fig. 2:**
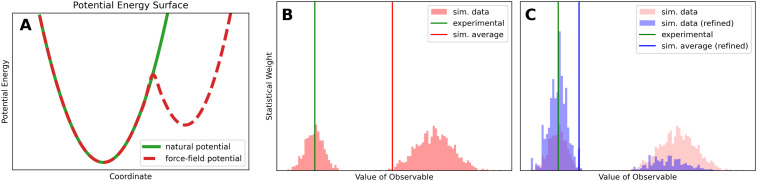
(A) A hypothetical natural potential with one minimum compared to a force field approximation with two minima. The second small valley can be described as faulty feature of the force field which leads to wrong estimates of observables. (B) The histogram shows simulated values of one hypothetical observable before reweighting. Different conformations of the ensemble yield different values for the observable. The hypothetical faulty force field of (A) introduces a second population on the right. These improper conformations shift the simulated expectation value (red line) to a higher value. (C) In addition to the data shown in (B), the reweighted histogram can be seen in blue. The weights of the right group, which is considered incorrect, are lowered while the weights in agreement with the experiment are increased. As a result, the simulated average (blue line) is now in better agreement with the experiment (green line).

In biophysical experiments the behavior of a measured molecule is determined by its potential-energy landscape (natural potential). This potential-energy surface, governed by nature's physics, is a computationally inaccessible potential which can only be approximated by the force field (or the quantum mechanical method). To illustrate this concept, imagine a hypothetical force field that accurately represents the natural potential except for one region. In this example, the force field potential includes an additional energy valley that does not exist in the natural potential (compare Fig. 2A).

Fig. 2B, illustrates the distribution of simulated values of a hypothetical observable for an unbiased ensemble. An experimental ensemble average could be measured (green line). The computational estimate of the same observable can be predicted by averaging over the samples of the simulation, shown as red line. In this example, the experimental value corresponds to the left population of the simulated observable. Due to force field errors (Fig. 2A), some samples of the simulation are likely overrepresented, shifting the computational ensemble average away from the experimental result. In this hypothetical example, the right population is an artifact of the force field, causing the simulated expectation value (red line) to be overestimated.

In general, there are two main approaches to address such miscalculated observables due to force field errors. Experimentally derived boundary conditions can be imposed during the simulation, to correct the force field for a specific system. Because these conditions are set *a priori*, they are baked into the trajectory, making later adjustments complicated and expensive. An *a priori* approach, to impose experimental restraints during the simulation, may guide the ensemble towards otherwise unsampled conformations, but bears the risk of getting stuck in a small amount of local minima due to too strong restraints, potentially leading to unintentional overfitting to the experimental observable.

Alternatively, ensemble reweighting can be used *a posteriori* to increase the impact of conformations that agree with the experiment, while reducing the impact of conformations that are in disagreement with the experiment. Reweighting methods yield new weights for the ensemble such that inappropriate conformations become insignificant. In our example, the refined simulated value of the observable can be seen in Fig. 2C (blue line) after the reweighting. Now the simulated average is in much better agreement with the experimental observable. This example already demonstrates key requirements necessary for the successful reweighting of conformational ensembles. The initial ensembles needs to be well-sampled, covering the entire relevant conformational space. In a second step, after the initial ensembles has been generated, the reweighting algorithm picks a sub-ensemble to better represent the experimental data by adjusting the statistical weights of the ensemble. As ensemble reweighting cannot generate new conformations that were not in the initial ensemble all relevant conformations must be sampled beforehand. An in-depth discussion on imposing boundary conditions *a priori* as compared to *a posteriori* reweighting can be found in Rangan *et al.*^40^

## 3 Reweighting algorithm

Over the course of years, several methods have been developed to integrate simulations with experimental data to further the understanding of biophysical processes. These methods can be divided into two main groups, depending on the optimization objective set. Maximum parsimony methods try to find a minimal^41–43^ or deliberately small^44–46^ ensemble in agreement in the data, for which multiple algorithms have been proposed. On the other hand entropy maximizing^29,47–53^ and Bayesian methods^54–62^ try to use as much of the initial information collected from MD simulations while balancing those with the experimental data. Maximum entropy methods may also be used to optimize force fields.^63^ Further reading beyond the scope of this work on the comparison of methods, including the maximum occurrence method,^64,65^ can be found with Medeiros Selegato *et al.*^66^ Additionally, a comprehensive overview about available methods has been collected by Bonomi *et al.*^67^

Regarding the nomenclature of methods, we understand the term maximum entropy methods as an umbrella term for a group of specific methods and implementations in which the initial ensembles are modified as little as possible given the conditions. This clearly separates maximum entropy methods from maximum parsimony methods, which maximally reduce the ensemble. The scope of this work focuses on the explanation and investigation of Bayesian ensemble refinement and the minimum relative entropy method, both commonly used methods within the maximum entropy umbrella term due to their closeness to the maximum entropy principle. A special case of the minimum relative entropy method, in which the initial weights are uniform, may also be referred to as entropy maximizing, as described in the appendix.

### 3.1 Bayesian ensemble refinement

Bayesian ensemble refinement has its foundations in Bayes' theorem which allows one to update the probability of an established hypothesis as new data becomes available. Accordingly, a method to update an existing model with new data allows for extensive opportunities to optimize conformational ensembles.^54,60^

Bayes’ theorem allows to calculate the conditional probabilities of events:4
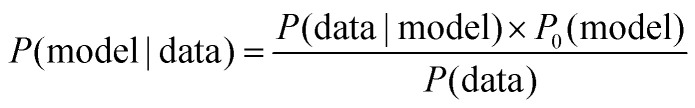
where *P*(model|data) is the posterior probability, *i.e.* the probability of the model, given the data. *P*(data|model) is the conditional probability to find data given the model. *P*_0_(model) is the prior probability and *P*(data) the marginal probability. The weights ***w*** of the conformational ensemble are the model and the measured and calculated observables ***O***^**exp**^ and ***O***^**calc**^ the data.

The prior probability *P*_0_(model) is the estimated probability of being correct before any data is observed. In the context of ensemble reweighting, the associated model parameters could be obtained from MD simulations. The conditional probability *P*(data|model) is a measure for the likelihood that the assumed model parameters can reproduce the observed data. The marginal probability can be interpreted as normalization constant such that the posterior probability qualifies as probability. It can be ignored in the case of an optimization problem where we search for the model that maximizes the posterior probability.

The basic formulation of Bayesian ensemble refinement sees the weight vector ***w*** as the model to describe the ensemble. As such, the method can be summarized as:

5 *P*(***w***|data) ∝ *P*(data|***w***) × *P*_0_(***w***)

To design an appropriate function that measures how well the model parameters explain the observed data, *P*(data|***w***) should have a maximum when simulated and observed data match each other. It may be interpreted as the likelihood that the data can be reproduced given the model weights ***w***. In the context of ensemble reweighting such a function can be designed as shown in eqn (6) if a Gaussian error can be postulated:6
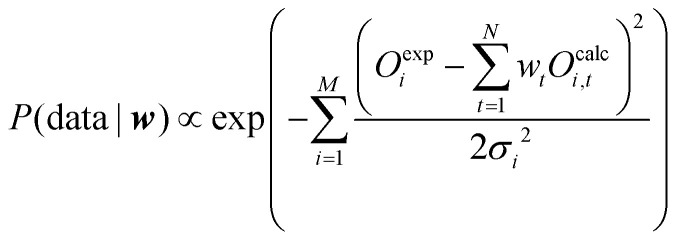
where *σ* is the standard deviation of the measured observable and *M* the number of observables.

For the prior probability of a model, we postulate that the model obtained from the unbiased simulation (***w***_**0**_) is the best representation of the true system. Thus, the probability to yield correct values for observables should be highest if ***w*** = ***w***_**0**_.^68^ A qualifying (but not normalized) function comparing ***w*** with ***w***_**0**_ can be found in the theta-scaled Kullback–Leibler divergence,^69^ which is equal to the relative entropy (eqn (7)) if the targeted distribution is normalized [ref. 70, p. 90] and theta is one:7
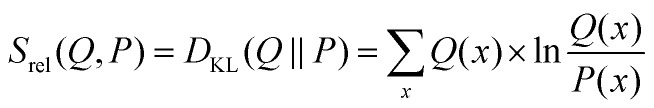

8 *P*_0_(***w***,***w***_**0**_) ∝ exp(−*θS*_rel_(***w***,***w***_**0**_))

where *S*_rel_ is the relative entropy with *S*_rel_ ≥ 0.0; *P* and *Q* probability distributions and *θ* a proportionality constant with *θ* ≥ 0.0.

To find the ideal model ***w***^**opt**^, the global maximum of the posterior probability (eqn (5)) needs to be found:9
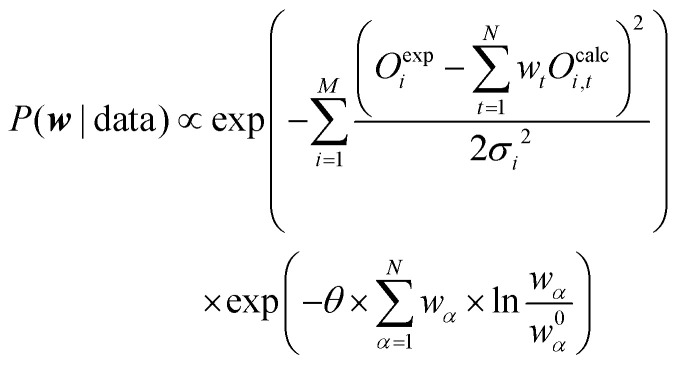


From eqn (9) the natural logarithm can be applied on both sides of the equation as the logarithm is a positive monotone transformation which does not alter the position of the extreme. After reordering the equation, the negative log posterior can be renamed to a cost function which leads to eqn (10):10
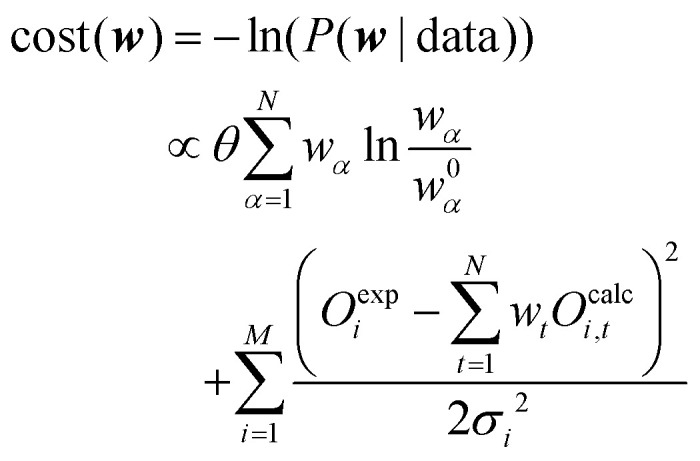


The minimum of the newly created cost function has to be found. The first term refers to the divergence to the initial distribution which should be small and the second term to the error to the experiment which also should be minimized:

11 optimize cost(*w*_0_, *w*_1_, …, *w*_*N*_) → min

The choice of *θ* is system specific and an expression of the quality of the initial distribution of weights. A large value of *θ* results in an optimization that stays very faithful to ***w***_**0**_ and accepts more significant violations in the data. A value of *θ* close to zero leads to a better agreement with the experimental data but ***w***_**0**_ is only of little relevance, which exposes the risk of overfitting.^54^

The second term of the cost function evaluates the error of the simulated observables ***O***^**calc**^ compared to the measured observables ***O***^**exp**^ and resembles closely the 
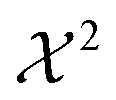
 distribution, except for a constant. The constant is utilized in some implementations while not in others. This leads to a change of scale of theta depending on the specific implementation. In both cases, the value of 
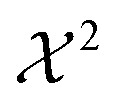
 quantifies the error between the experiment and the simulation (weighted by *w*_*t*_).^60^12
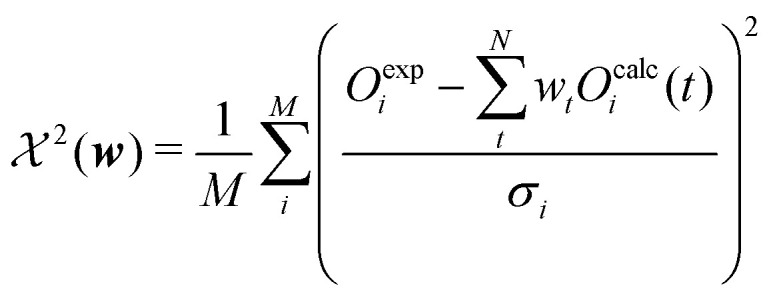


Eqn (12) may be adjusted if the measured observable is not a scalar with a specific value but a range of valid results. In the case of NOE analysis the measured distance of a proton pair is described by a range of values enclosed by a lower and upper bound.^71^ For reweighting lower and upper bounds are set independently as one-sided limits; therefore implementations must make sure that only violated bounds contribute to eqn (12).

### 3.2 The minimum relative entropy (MRE) method

The minimum relative entropy method allows to find a set of weights that minimizes the relative entropy compared to the ensemble obtained from a MD simulation while fulfilling predefined conditions.

In addition to the relative entropy (*S*_rel_, eqn (7), ref. 69) it is common to define two additional types of entropy that depend on one or two probability distributions (*Q* and *P*):^72,73^13
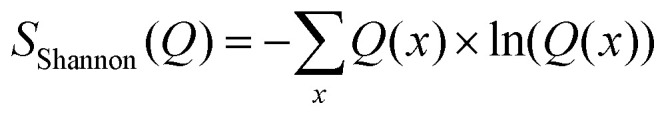
14
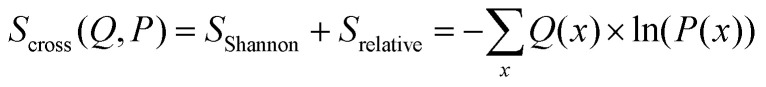


The maximum entropy method introduced by Jaynes^74,75^ allows to find a probability distribution that is in agreement with external conditions while preserving maximal entropy given the conditions.^76^ The relative entropy can be interpreted as the information lost when using distribution *Q*(***x***) as an approximation of distribution *P*(***x***). If minimized, the distribution *Q*(***x***) can be assumed to be the distribution that meets all necessary conditions while requiring minimal additional information.^77^

The Shannon entropy (eqn (13)) reaches its maximum when the probability distribution is uniform.^78^ This property of the Shannon entropy explains why most methods in conformational ensemble reweighting that try to preserve the initial ensemble generated with MD are called maximum entropy methods. It can be shown (Appendix A.1) that the maximum entropy method can be a special case of the minimum relative entropy method if the weights ***w***_**0**_ are uniform [ref. 79, pp. 291–292].

The relative entropy (also called Kullback–Leibler divergence, eqn (7)) is the difference between Shannon- and cross-entropy (eqn (14)) and a metric to evaluate the similarity of two probability distributions. If both discrete probability distributions *Q* and *P* are equal, the relative entropy is zero. The relative entropy is positive and increases with diverging distributions *Q* and *P* [ref. 70, p. 90]. An important property of the KL-divergence is it being not symmetric and failing to satisfy the triangle inequality, thus making it a divergence between the distributions and not a distance.^80,81^

From eqn (14) follows an alternative notation of the relative entropy:15
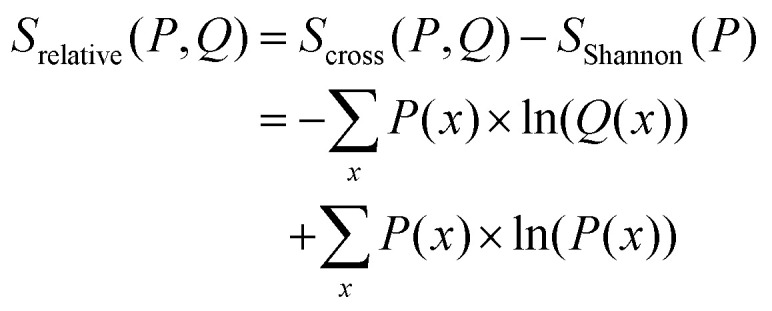


Due to the non-symmetry of the relative entropy a distinction between a forward case and a reversed case can be made (see ref. 80, pp. 71–74 and ref. 81–85). In the context of optimization methods, one of the two distributions is kept constant (*P*(*x*), reference distribution) while the other (*Q*_v_(*x*), approximated distribution) is being learned and therefore dependent on the optimization parameter.^86^

Forward KL-divergence (eqn (16))16
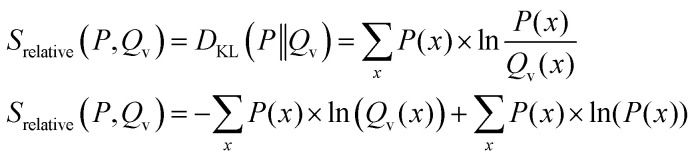


From eqn (16) it becomes apparent that the contribution of the Shannon entropy is independent from the variable distribution *Q*_v_(*x*) and doesn't influence the minimization of the relative entropy. Therefore, the minimization of the relative entropy in the forward formulation is equal to the minimization of the cross-entropy and often referred to as the minimum cross entropy method in literature.

It can be shown that the forward formulation of the KL-divergence is closely related to the maximum likelihood *P*(*x*) is chosen (Appendix A.2).

Reversed KL-divergence (eqn (17))17
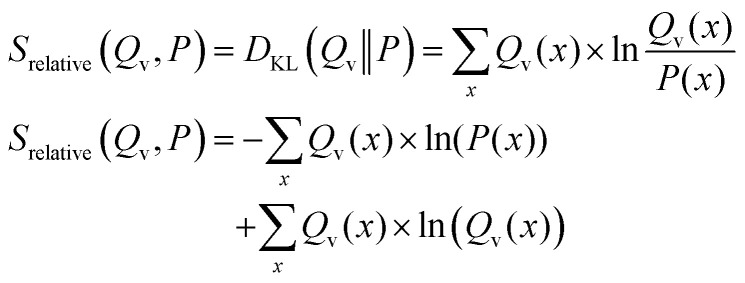


In contrast to the forward formulation, the contribution of the Shannon entropy to the relative entropy is variable and cannot be ignored when using the reversed KL-divergence as loss function.

**Fig. 3:**
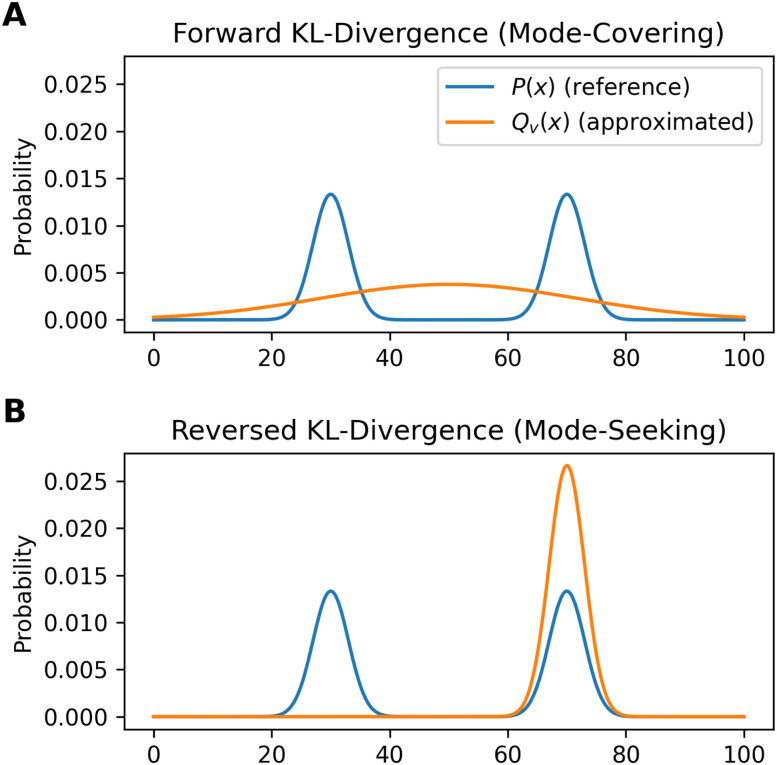
An example to demonstrate the different behavior of forward and reversed KL-divergence loss in the optimization of systems. The reference distribution (*P*(*x*), blue) consists of two Gaussian functions summed up (*μ*_1_ = 30; *μ*_2_ = 70; *σ*_1,2_ = 3) and normalized to one. The optimized distribution (*Q*_v_(*x*), orange) consists of one normalized Gaussian function with two optimized parameters (*μ*,*σ*). The loss function of the optimization is the Kullback–Leibler divergence which is minimized. In case of the mode-seeking behavior, two solutions are possible as both the left and right peak may be approximated by *Q*_v_(*x*). In this example, the choice of the initial guess of *μ* decides which peak gets approximated.

In practice, differences become relevant when systems with a low number of independent parameters are optimized. Fig. 3 shows an example illustrating the influence of the chosen loss function on the fitted distribution. The bimodal reference distribution *P*(*x*) in blue is to be approximated by a single Gaussian optimised distribution, *Q*_v_(*x*). An optimization using the forward KL-divergence is referred to as mode-covering (inclusive) and leads to a single broad distribution. The reversed KL-divergence optimization is called a mode seeking (exclusive) approach and leads to the selection of a single signal in the reference distribution.^83,84,86^ The relation of the reverse formulation of the minimum relative entropy method to the maximum entropy method given a uniform target distribution is shown in Appendix A.1.

The directionality of KL-divergence based loss functions is an important theoretical consideration when designing algorithms in data science. While Fig. 3 shows an example specifically designed to present the directionality of the loss function, its effect during reweighting of ensembles is more subtle. Nevertheless, we see minor differences when optimizing the same data using the same strength of optimization *θ*. In our work Stöckelmaier *et al.*^87^ we created a validation system for ensemble refinement using the small dialanine peptide. While a quantitative assessment of the algorithm presented here is beyond the scope of this work, we would like to refer to the Appendix A.3 showing the impact of the loss function directionality of Bayesian ensemble refinement. This and other comparisons in our previous work^87^ indicate, that the effect of the directionality is not dramatic but noticeable when refining conformational ensembles.

#### 3.2.1 Solution of the minimum relative entropy problem (reversed case) in the context of conformational ensemble refinement

Ensemble refinement using the minimum relative entropy method is commonly applied to ensembles from MD simulations. The optimization strategy presented here is used to solve the minimum relative entropy problem in its reversed formulation. Leveraging Lagrange multipliers it reduces the number of optimized parameters to the number of observables, allowing for efficient reweighting. Even if the solution strategy presented here is not the only one available, its application in established methods such as Bottaro *et al.*^52^ makes it particularly relevant to discuss in detail.

An initial distribution of weights (***w***^**0**^) is typically available from MD. It may be uniform if the data comes straight from MD or non-uniform if the data is reduced by clustering the conformational ensemble or obtained from biased ensemble methods like replica exchange MD. Both cases can be treated with the minimum relative entropy method. If the initial ensemble has been reduced by clustering, each calculated observable representing the cluster should, by itself, be an ensemble-average of the cluster.^88^ To optimize the conformational ensemble, the set of weights ***w***_**opt**_ has to be found that minimizes the relative entropy *S* (eqn (18)) in reference to ***w***_**0**_:18
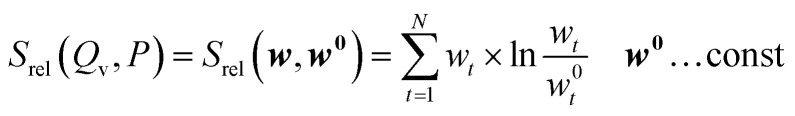


However, the minimization should be performed obeying two boundary conditions. The first represents the condition that the calculated and experimental ensemble averages of the observables should match:19
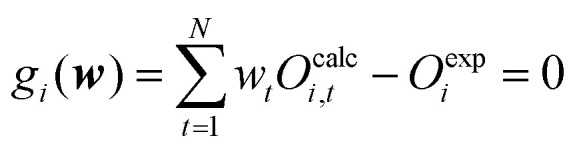


The second condition is a reformulation of eqn (3) and enforces that the updated probability distribution remains normalized:20
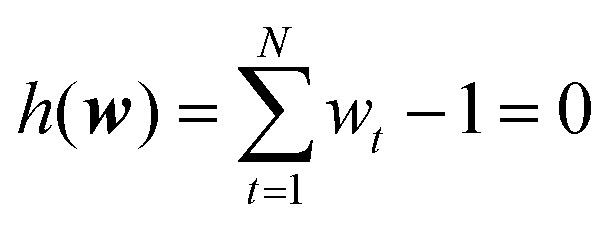


An optimization under the constraints given by eqn (19) and (20) can be solved using Lagrange-multipliers, *λ*_*i*_ and *μ*. The sign in front of each condition term does not influence the solution.21
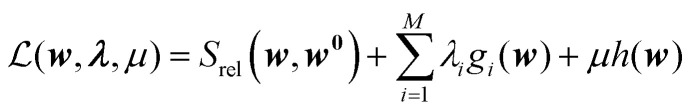


The partial derivative of eqn (21) with respect to each vector element *w*_*t*_ is taken:22
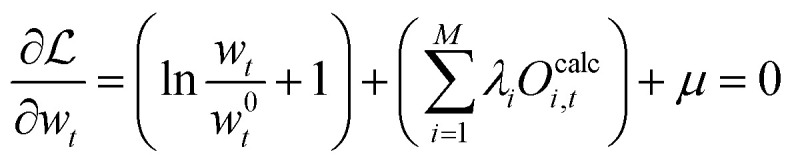


This equation can be rearranged and we can define *λ*_0_ as:

23 *λ*_0_ := 1 + *μ*

such that24
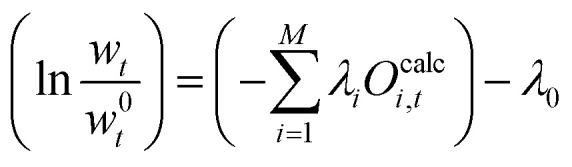
25
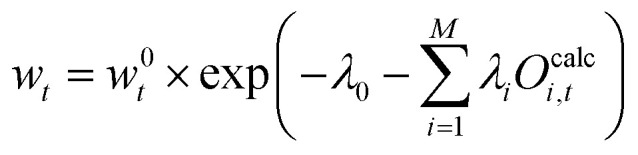
26
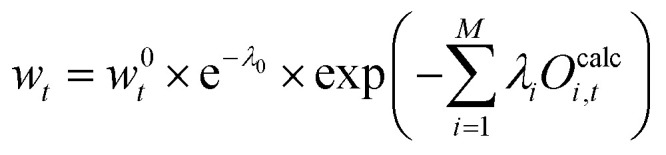


The term e^−*λ*_0_^ should be interpreted as normalization term. The value of e^−*λ*_0_^ can be obtained using the condition (20) which leads to eqn (27):27
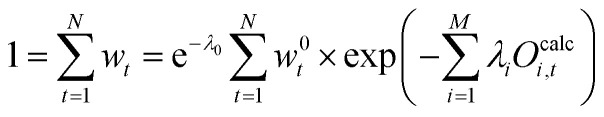


We define a partition function, *Z*:28
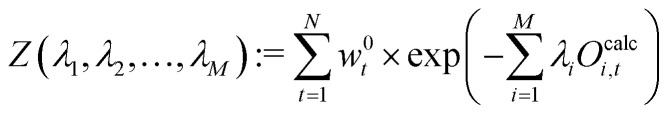
which can be determined from eqn (27):29
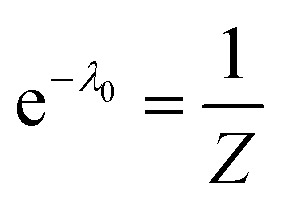


Combining eqn (26) and (29) the reweighted probabilities can be calculated:30
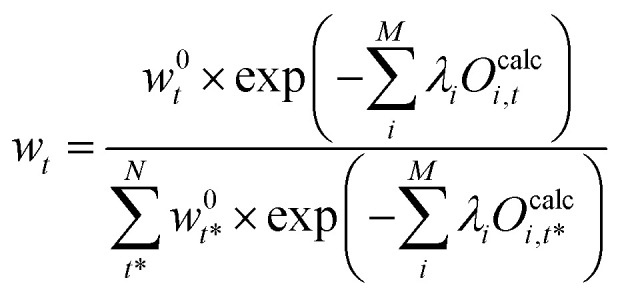


Eqn (30) connects the optimal weights for the *N* conformations in the ensemble to the Lagrange multipliers, *λ*_*i*_ for each of the *M* observables. This significantly reduces the dimensionality of the optimization problem, but solving a *M*-dimensional optimization problem still remains a difficult task. To calculate the vector ***λ*** it is possible to turn the problem into an easier optimization problem using the Lagrangian duality formalism. A solution is described in ref. 48, 89 and 90 and used in ref. 29, 91 and 92.

The concave Lagrangian dual *Γ*(***λ***,*μ*) is introduced as a function of the primal optimization problem 
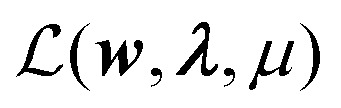
:31
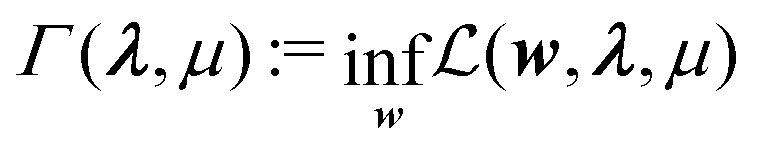


Remember that the vector ***w***_**opt**_ should fulfill conditions (19) and (20). Accordingly, for the optimal solution, the condition terms of eqn (21) become zero. To calculate the infimum of the Lagrangian dual 
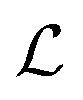
, eqn (24) gets substituted into the entropy term of eqn (21) which leads to eqn (32):32
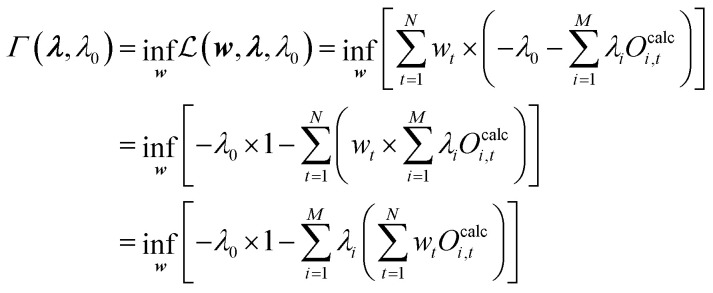


Replacing −*λ*_0_ with eqn (29) then leads to:33
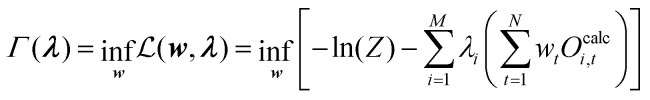


From the initial condition (19) it is defined that 
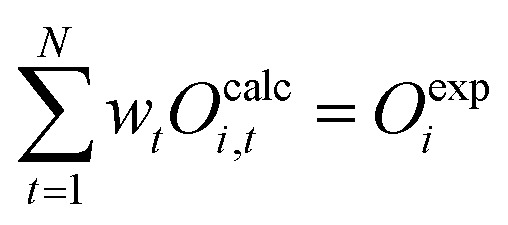
.34
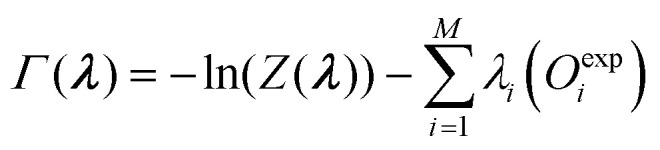


To determine the optimal Lagrangian multipliers, the maximum of the concave Lagrangian dual is determined (sup_***λ***_*Γ*(***λ***)). The function *Γ* (eqn (34)) should be maximized without constraints.

##### Treatment of experimental error in the MRE method

Until now, the resulting weights are constrained to exactly recover the experimental average (eqn (19)). In many cases, solving the fully constrained formulation of the problem would lead to unwarranted overfitting as both the simulated observables and the experimental data contain some level of error which needs to be taken into account. Error treatment as shown below was described by Cesari *et al.*,^29,91^ introducing:

35 *O*^exp^_*i*_ + 〈*ε*_*i*_〉 = 〈*O*^calc^_*i*_〉

where *ε*_*i*_ is the expected total of all errors for observable *i*.

Instead of the original condition (19) an error corrected condition 
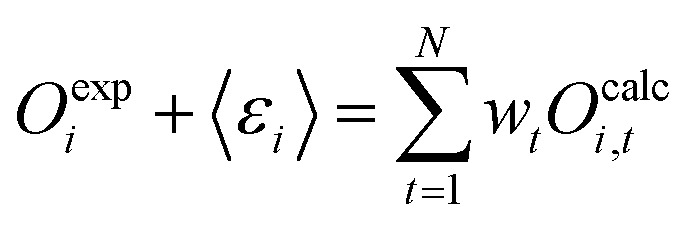
 can be used. Therefore, the modified *Γ*-function for optimization problems including error is obtained:36
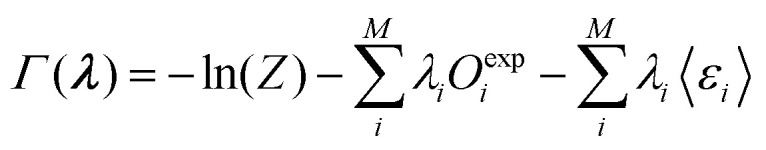


Cesari *et al.*^29^ further describe the methodology of treating a Gaussian shaped error with preassigned variance. The third term in eqn (36), describing the error, becomes:37
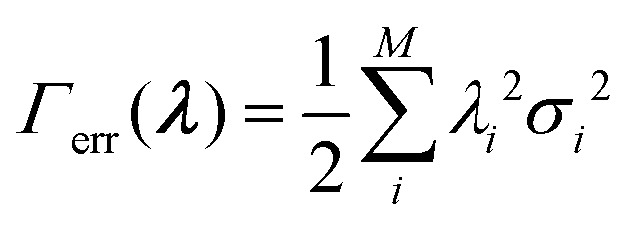


Finally, a proportionality constant *θ* is introduced which defines the influence of the error *ε*_*i*_ on the optimization. A choice of a large *θ* indicates that larger error are tolerated. If Gaussian shaped errors are assumed, eqn (38) should be maximized:38
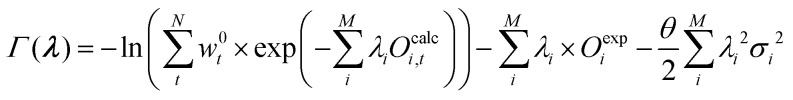


#### 3.2.2 Solution of the minimum relative entropy problem (forward case) in the context of conformational ensemble refinement

After the successful creation of the *Γ*-function to solve the minimum relative entropy problem in its reversed case, it is of interest if the same logic can also be applied to the forward case.

According to the definition of the forward KL-divergence, we define the relative entropy as39
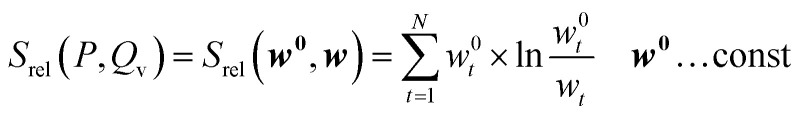


The Lagrangian function is set up similar to (21) but with the alternative entropy term. The partial derivative of the modified Lagrangian is taken and set to zero.40
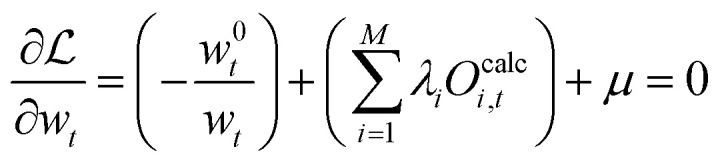


Here, a significant difference to eqn (22) can be seen as the fraction *w*^0^_*t*_/*w*_*t*_ in the equation is outside of a logarithm. The solution for the forward direction can still be formulated in terms of an optimization of the Langrange multipliers (*via*eqn (41)) but solving the problem as described previously is difficult, as the 'normalization constant’ *μ* cannot be calculated easily.41
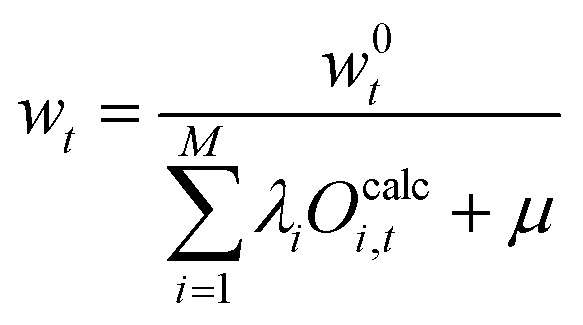


In practice, the reverse formulation of the KL-divergence remains more accessible when using a Lagrangian solution strategy. It is the regular choice as loss function even though the mode-covering behavior of the forward case remains interesting for the optimization of molecular ensembles. Non-Lagrangian solution strategies to optimize ensembles using the forward case remain attractive and can be seen as a further area of research. As a basic solution, Bayesian ensemble refinement described in Section 3.1 can easily be modified to apply both the forward and the reversed KL-divergence.

### 3.3 Estimation of the hyper-parameter *θ*

Both the direct optimization of the weights using Bayesian ensemble refinement in Section 3.1 as well as the (error-aware) indirect optimization using the maximum entropy formalism in Section 3.2 uses a hyper parameter theta (*θ*), to set the strength of the optimization. It can be freely tuned taking any positive value and sets the balance between faithfulness to ***w***^**0**^ and reduction in error compared to the experimental data. The optimal choice of *θ* avoids overfitting of the data while allowing sufficient reweighting. As *θ* is difficult to set in advance, a strategy to find a suitable value for theta has to be introduced.

**Fig. 4:**
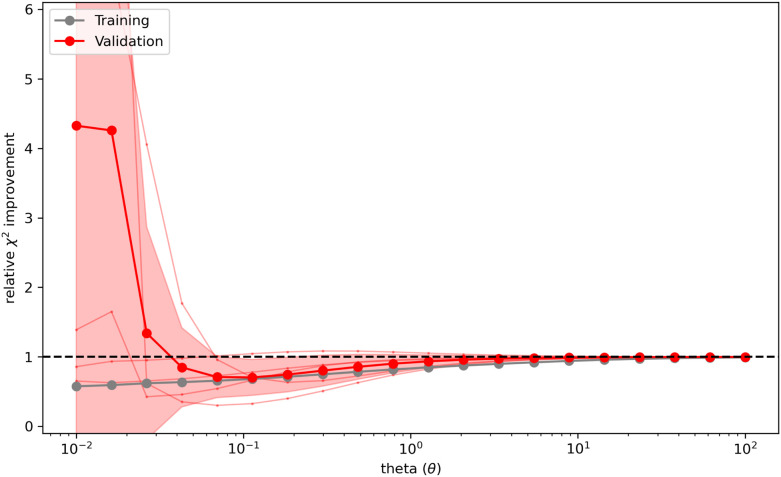
Cross validation may be used to prevent overfitting of the data. The *x*-axis shows *θ* while the *y*-axis shows the relative error between experiment and simulation (relative 
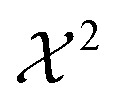
 improvement). Gray represents the error against the training data while red represents the error against the validation data. To reweight an ensemble, *θ* should be chosen to represent the minimum of the curve. If no clear minimum is found, it should be avoided to set *θ* in a range of values that lead to an increase of the relative 
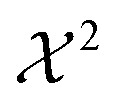
 improvement. A too small *θ* likely leads to overfitting of the data.

Bottaro *et al.*^92^ describes a five-fold cross-validation to estimate the optimal value of *θ* for their implementation of the reversed maximum entropy approach. The observables and the conformational ensemble are split into a training and validation data-set. The training set is used to calculate the optimized weights ***w*** while the validation set uses these weights to calculate the relative 
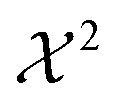
 improvement (
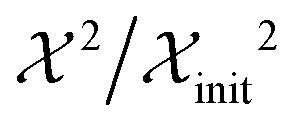
, where 
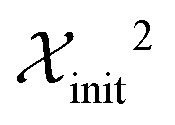
 is calculated using the initial weights ***w***_**0**_) as a validation score. This process is repeated for a set of different *θ* values. If a set of weights improves not only the agreement between simulation and experiment in regard to the fitted observables, but also in regard to previously unknown ones from the validation set, a validation score below one is calculated. It may be interpreted as the ability to find a set of weights compatible with the prior information, simulated and experimental data that is likely an improvement over the initial set of weights. On the other hand, a validation score over one may be interpreted as the inability to find a set of weights in agreement with prior information, simulated and experimental data that is an improvement over the initial set of weights in regard to previously unknown observables. Thus, it may indicate overfitting of the data. A plot (Fig. 4) showing the relative 
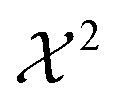
 improvement as function of *θ* is used to tune the strength of the optimization. In the best case, a well behaving curve with little uncertainty is shown, indicating an ideal choice of *θ* at the minimum of the curve. In practice, the curve often shows substantial levels of noise and lacks an obvious minimum but shows a steep increase of the relative 
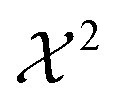
 improvement at low theta values. In this case, it may be reasonable to choose a value of *θ* just before the steep increase in slope manifests. To confirm the plausibility of the chosen *θ*, the resulting ensemble after reweighting should be checked manually to confirm that the new ensemble remains plausible, both in size and conformations.

Alternatively, it is also possible to tune the strength of optimization such that the optimized ensemble evaluates to an error estimate of 
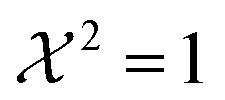
.^60,93,94^ A 
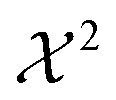
-value of one quantifies that the average error of the ensemble is equal to the sum of uncertainty from experiment and simulation. While this approach is straightforward at first glance, it assumes that the uncertainty from simulation and experimental measurement is additive and well characterized. In many practical application, as in our recent work,^87^ both the uncertainty from simulation and experiment is guessed, making the absolute value of 
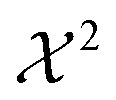
 a reasonable indicator but difficult to use as a conclusive criterion.

### 3.4 Mutual similarities and differences

Bayesian ensemble refinement and the minimum relative entropy method show similarities and differences in regard to each other. Their relationship with the maximum entropy principle is mutual but their specific properties show interesting differences. Additional information about the connection between Bayesian probability theory and maximum entropy methods can be found in the literature, where the work of Jaynes^95^ and Skilling^96^ should be noted.

#### 3.4.1 Error regularization

The quantification of error between experiment and simulation is central when performing ensemble reweighting. The minimum relative entropy method enforces the minimization of the linear error on a per-observable basis due to the Lagrangian formalism to solve the optimization problem. The classical Bayesian approach regulates the optimization using a global 
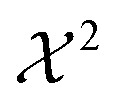
-like error, allowing for a compensation of errors. In the minimum relative entropy method *θ* scales the error constraint between simulation and experiment while in the classical Bayesian approach the influence of error and entropy regularization gets balanced.

#### 3.4.2 Entropy regularization

To ensure that the optimized ensemble weights stay faithful to the initial simulation, both discussed methods use the KL-divergence to govern the similarity between optimized and initial ensemble. The KL-divergence offers both a forward and a reversed direction. While the minimum relative entropy method uses the reversed approach, Bayesian ensemble refinement can easily be applied in both directions.

#### 3.4.3 Calculation of statistical weights

The classical Bayesian approach calculates the optimized statistical weights of the ensemble directly using a cost function dependent on ***w***. In contrast, the minimum relative entropy method first optimizes a proxy vector (***λ***) with a significant lower number of variables to then backcalculate the weights from the proxy.

## 4 Conclusions

Maximum entropy based ensemble optimization shows promising properties to allow the integration of experimental and simulated data. To gain a better understanding of flexible and intrinsically disordered proteins, the combination of experimental techniques like liquid state NMR and molecular dynamics (MD) simulations seems essential. Contrary to globular proteins, flexible proteins cannot be described using just a single (crystal) structure but require the creation of a conformational ensemble. Each conformer within the ensemble is associated with a statistical weight quantifying their importance to the ensemble. The calculation of these weights is non-trivial and requires computational studies. MD allows to sample appropriate weights but is prone to inaccuracies, especially with disordered proteins where the sampling may be expected to be incomplete. Maximum entropy based ensemble optimization allows us to adapt these initial weights, such that the resulting ensemble remains close to the MD-simulation and agrees with the experimental data.

In the last decade, numerous implementations of maximum entropy methods have been developed and applied. The theoretical foundation behind the methods is based on the established information theory by Claude Shannon. While the theory behind ensemble refinement is solid and well established, most methods work as black-box optimizer for many users. In this work, we focused on the foundation of the technique to promote a broader understanding of the methods as we believe this is important to allow for proper interpretation of the refined conformational ensembles. We want to emphasize that reweighting methods require well curated data, both simulated, experimental and in regard to prior weights. Ill curated data used during the process of reweighting may lead to misleading findings that are difficult to spot and may promote incorrect findings. In summary, however, it can be stated that reweighting works well if used carefully with well curated data. Maximum entropy methods show a solid theoretical foundation and promising properties to integrate simulated and experimental data, allowing new and exciting insights into molecular behavior.

## Author contributions

JS reviewed and summarized the theoretical foundation of maximum entropy methods and wrote the manuscript. CO acted as supervisor, organized the funding of the project, edited and reviewed the writing of the manuscript. All authors have read and agreed to the published version of the manuscript.

## Conflicts of interest

The authors declare that there are no conflicts of interest.

## Figures and Tables

**Fig. 5:**
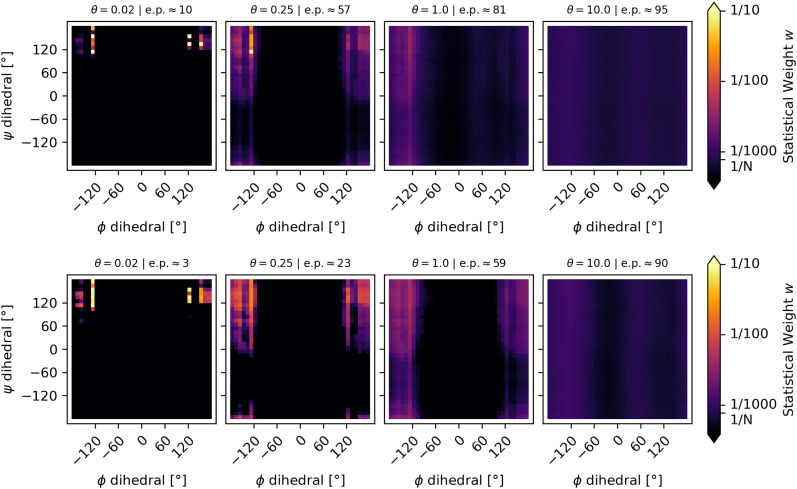
The loss-function of Bayesian ensemble refinement allows for an easy implementation of both the forward and reversed direction of the KL-divergence. Using the dialanine system with equipotential (uniform) initial weights as presented in ref. 87 to test ensemble refinement, the directional dependence of Bayesian ensemble refinement was tested. The top row shows the result of the reweighting using the forward (mode-covering) direction with four different values of theta tested. The second row shows the same system with the same *θ*-values tested using the reversed (mode-seeking) direction.

## Data Availability

No primary research results, software or code have been included and no new data were generated or analysed as part of this review.

## Appendices

### Appendix: A

#### A.1 Maximum entropy – minimum relative entropy^79^

Consider the reversed formulation of the Kullback–Leibler divergence:

42

In the case of a uniform distribution *P*(*x*) this leads to:

43

44

45

46 *D*_KL_(*Q*_v_‖*P*) = −(*S*_Shannon_) + const

As ln(*N*) is a constant, optimizing *Q*_v_ with respect to minimizing the relative entropy is equivalent to maximizing the Shannon entropy if the target distribution is uniform. Thus, maximum entropy optimizations are closely related to the minimum relative entropy optimizations in its reversed formulation.

#### A.2 Log probabilities – minimum relative entropy^81^

Consider the forward formulation of the Kullback–Leibler divergence:

47

In the case of a uniform distribution *P*(*x*) this leads to:

48

49

50

51

The first term of eqn (50) is constant. In consequence, the close relation between the relative entropy minimization in its forward formulation and the negative log-likelihood minimization is shown if a uniform distribution *P*(*x*) is chosen.

#### A.3 The directional dependence of Bayesian ensemble refinement

Fig. 5 shows the weights of the reweighted dialanine system which is described in ref. 87. To interpret the results, the ensemble preservation (e.p.) metric as introduced in ref. 87 is used. In a simplified way, the e.p. can be understood such, that a preservation of 100 indicates that all conformations remain in the ensemble; a preservation of 33 indicates that only one third of initial conformations still contribute to the optimized ensemble.

Column two (*θ* = 0.25) demonstrates the subtle differences between the directions. While in general the same regions get populated, the ensemble preservation of the forward (mode-covering) direction remains higher with (on average) lower weights in the preferred β-sheet region.

## References

[cit1] Fischer E. (1894). Einfluss der Configuration auf die Wirkung der Enzyme. Ber. Dtsch. Chem. Ges..

[cit2] Anfinsen C. B. (1973). Principles that Govern the Folding of Protein Chains. Science.

[cit3] Uversky V. N., Kulkarni P. (2021). Intrinsically disordered proteins: Chronology of a discovery. Biophys. Chem..

[cit4] Ward J., Sodhi J., McGuffin L., Buxton B., Jones D. (2004). Prediction and Functional Analysis of Native Disorder in Proteins from the Three Kingdoms of Life. J. Mol. Biol..

[cit5] Kulkarni P., Leite V. B. P., Roy S., Bhattacharyya S., Mohanty A., Achuthan S., Singh D., Appadurai R., Rangarajan G., Weninger K., Orban J., Srivastava A., Jolly M. K., Onuchic J. N., Uversky V. N., Salgia R. (2022). Intrinsically disordered proteins: Ensembles at the limits of Anfinsen's dogma. Biophys. Rev..

[cit6] Trovato F., Trylska J., Bond P. J., Wolynes P. G. (2021). Front. Mol. Biosci..

[cit7] Wang H., Xiong R., Lai L. (2023). Rational drug design targeting intrinsically disordered proteins. Wiley Interdiscip. Rev.:Comput. Mol. Sci..

[cit8] Piovesan D., Del Conte A., Mehdiabadi M., Aspromonte M. C., Blum M., Tesei G., von Bülow S., Lindorff-Larsen K., Tosatto S. C. E. (2025). MOBIDB in 2025: integrating ensemble properties and function annotations for intrinsically disordered proteins. Nucleic Acids Res..

[cit9] Chebaro Y., Ballard A. J., Chakraborty D., Wales D. J. (2015). Intrinsically Disordered Energy Landscapes. Sci. Rep..

[cit10] Viegas R. G., Martins I. B. S., Leite V. B. P. (2024). Understanding the Energy Landscape of Intrinsically Disordered Protein Ensembles. J. Chem. Inf. Model..

[cit11] Jensen M. R., Zweckstetter M., Huang J.-R., Blackledge M. (2014). Exploring Free-Energy Landscapes of Intrinsically Disordered Proteins at Atomic Resolution Using NMR Spectroscopy. Chem. Rev..

[cit12] Scheek R. M. , Torda A. E., Kemmink J. and van Gunsteren W. F., in Computational Aspects of the Study of Biological Macromolecules by Nuclear Magnetic Resonance Spectroscopy, ed. J. C. Hoch, F. M. Poulsen and C. Redfield, Springer US, Boston, MA, 1991, pp. 209–217

[cit13] Frauenfelder H., Sligar S. G., Wolynes P. G. (1991). The Energy Landscapes and Motions of Proteins. Science.

[cit14] Lindorff-Larsen K., Best R. B., DePristo M. A., Dobson C. M., Vendruscolo M. (2005). Simultaneous determination of protein structure and dynamics. Nature.

[cit15] Fisher C. K., Stultz C. M. (2011). Constructing ensembles for intrinsically disordered proteins. Curr. Opin. Struct. Biol..

[cit16] Borodina Y. V., Bolton E., Fontaine F., Bryant S. H. (2007). Assessment of Conformational Ensemble Sizes Necessary for Specific Resolutions of Coverage of Conformational Space. J. Chem. Inf. Model..

[cit17] Liu W., Liu X., Zhu G., Lu L., Yang D. (2018). A Method for Determining Structure Ensemble of Large Disordered Protein: Application to a Mechanosensing Protein. J. Am. Chem. Soc..

[cit18] Schwede T., Kopp J., Guex N., Peitsch M. C. (2003). SWISS-MODEL: An automated protein homology-modeling server. Nucleic Acids Res..

[cit19] Baek M., DiMaio F., Anishchenko I., Dauparas J., Ovchinnikov S., Lee G. R., Wang J., Cong Q., Kinch L. N., Schaeffer R. D., Millán C., Park H., Adams C., Glassman C. R., DeGiovanni A., Pereira J. H., Rodrigues A. V., van Dijk A. A., Ebrecht A. C., Opperman D. J., Sagmeister T., Buhlheller C., Pavkov-Keller T., Rathinaswamy M. K., Dalwadi U., Yip C. K., Burke J. E., Garcia K. C., Grishin N. V., Adams P. D., Read R. J., Baker D. (2021). Accurate prediction of protein structures and interactions using a three-track neural network. Science.

[cit20] Jumper J., Evans R., Pritzel A., Green T., Figurnov M., Ronneberger O., Tunyasuvunakool K., Bates R., Žídek A., Potapenko A., Bridgland A., Meyer C., Kohl S. A. A., Ballard A. J., Cowie A., Romera-Paredes B., Nikolov S., Jain R., Adler J., Back T., Petersen S., Reiman D., Clancy E., Zielinski M., Steinegger M., Pacholska M., Berghammer T., Bodenstein S., Silver D., Vinyals O., Senior A. W., Kavukcuoglu K., Kohli P., Hassabis D. (2021). Highly accurate protein structure prediction with AlphaFold. Nature.

[cit21] Lewis S. , Hempel T., Jiménez-Luna J., Gastegger M., Xie Y., Foong A. Y. K., Satorras V. G., Abdin O., Veeling B. S., Zaporozhets I., Chen Y., Yang S., Schneuing A., Nigam J., Barbero F., Stimper V., Campbell A., Yim J., Lienen M., Shi Y., Zheng S., Schulz H., Munir U., Tomioka R., Clementi C. and Noé F., Scalable emulation of protein equilibrium ensembles with generative deep learning, bioRxiv, 2025, preprint 10.1101/2024.12.05.626885 40638710

[cit22] Cagiada M. , Thomasen F. E., Ovchinnikov S., Deane C. M. and Lindorff-Larsen K., AF2: Predicting protein side-chain rotamer distributions with AlphaFold2, bioRxiv, 2025, preprint 10.1101/2025.04.16.649219

[cit23] Monteiro da Silva G., Cui J. Y., Dalgarno D. C., Lisi G. P., Rubenstein B. M. (2024). Highthroughput prediction of protein conformational distributions with subsampled AlphaFold2. Nat. Commun..

[cit24] Sala D., Engelberger F., Mchaourab H. S., Meiler J. (2023). Modeling conformational states of proteins with AlphaFold. Curr. Opin. Struct. Biol..

[cit25] Piana S., Lindorff-Larsen K., Shaw D. E. (2011). How Robust Are Protein Folding Simulations with Respect to Force Field Parameterization?. Biophys. J..

[cit26] Lindorff-Larsen K., Piana S., Dror R. O., Shaw D. E. (2011). How Fast-Folding Proteins Fold. Science.

[cit27] Piana S., Lindorff-Larsen K., Shaw D. E. (2012). Protein folding kinetics and thermodynamics from atomistic simulation. Proc. Natl. Acad. Sci. U. S. A..

[cit28] Kang W., Jiang F., Wu Y.-D. (2022). How to strike a conformational balance in protein force fields for molecular dynamics simulations?. Wiley Interdiscip. Rev.: Comput. Mol. Sci..

[cit29] Cesari A., Reißer S., Bussi G. (2018). Using the Maximum Entropy Principle to Combine Simulations and Solution Experiments. Computation.

[cit30] Thomasen F. E., Lindorff-Larsen K. (2021). Conformational ensemble of intrinsically disordered proteins and flexible multidomain proteins. Biochem. Soc. Trans..

[cit31] Czaplewski C., Gong Z., Lubecka E. A., Xue K., Tang C., Liwo A. (2021). Recent Developments in Data-Assisted Modeling of Flexible Proteins. Front. Mol. Biosci..

[cit32] Gama Lima Costa R., Fushman D. (2022). Reweighting methods for elucidation of conformation ensembles of proteins. Curr. Opin. Struct. Biol..

[cit33] Ozenne V., Schneider R., Yao M., Huang J.-R., Salmon L., Zweckstetter M., Jensen M. R., Blackledge M. (2012). Mapping the Potential Energy Landscape of Intrinsically Disordered Proteins at Amino Acid Resolution. J. Am. Chem. Soc..

[cit34] van Gunsteren W. F., Allison J. R., Daura X., Dolenc J., Hansen N., Mark A. E., Oostenbrink C., Rusu V. H., Smith L. J. (2016). Deriving Structural Information from Experimentally Measured Data on Biomolecules. Angew. Chem., Int. Ed..

[cit35] Grutsch S., Brüschweiler S., Tollinger M. (2016). NMR Methods to Study Dynamic Allostery. PLoS Comput. Biol..

[cit36] Camacho-Zarco A. R., Schnapka V., Guseva S., Abyzov A., Adamski W., Milles S., Jensen M. R., Zidek L., Salvi N., Blackledge M. (2022). NMR Provides Unique Insight into the Functional Dynamics and Interactions of Intrinsically Disordered Proteins. Chem. Rev..

[cit37] Tropp J. (1980). Dipolar relaxation and nuclear Overhauser effects in nonrigid molecules: The effect of fluctuating internuclear distances. J. Chem. Phys..

[cit38] Daura X., Antes I., van Gunsteren W. F., Thiel W., Mark A. E. (1999). The effect of motional averaging on the calculation of NMR-derived structural properties. Proteins: Struct., Funct., Bioinf..

[cit39] Zagrovic B., van Gunsteren W. F. (2006). Comparing atomistic simulation data with the NMR experiment: How much can NOEs actually tell us?. Proteins.

[cit40] Rangan R., Bonomi M., Heller G. T., Cesari A., Bussi G., Vendruscolo M. (2018). Determination of Structural Ensembles of Proteins: Restraining *vs.* Reweighting. J. Chem. Theory Comput..

[cit41] Pelikan M., Hura G. L., Hammel M. (2009). Structure and flexibility within proteins as identified through small angle X-ray scattering. Gen. Physiol. Biophys..

[cit42] Berlin K., Castañeda C. A., Schneidman-Duhovny D., Sali A., Nava-Tudela A., Fushman D. (2013). Recovering a Representative Conformational Ensemble from Underdetermined Macromolecular Structural Data. J. Am. Chem. Soc..

[cit43] Ihms E. C., Foster M. P. (2015). MESMER: minimal ensemble solutions to multiple experimental restraints. Bioinformatics.

[cit44] Chen Y., Campbell S. L., Dokholyan N. V. (2007). Deciphering Protein Dynamics from NMR Data Using Explicit Structure Sampling and Selection. Biophys. J..

[cit45] Nodet G., Salmon L., Ozenne V., Meier S., Jensen M. R., Blackledge M. (2009). Quantitative Description of Backbone Conformational Sampling of Unfolded Proteins at Amino Acid
Resolution from NMR Residual Dipolar Couplings. J. Am. Chem. Soc..

[cit46] Bernadó P., Mylonas E., Petoukhov M. V., Blackledge M., Svergun D. I. (2007). Structural Characterization of Flexible Proteins Using Small-Angle X-ray Scattering. J. Am. Chem. Soc..

[cit47] Różycki B., Kim Y. C., Hummer G. (2011). SAXS Ensemble Refinement of ESCRT-III CHMP3 Conformational Transitions. Structure.

[cit48] Pitera J. W., Chodera J. D. (2012). On the Use of Experimental Observations to Bias Simulated Ensembles. J. Chem. Theory Comput..

[cit49] Boomsma W., Ferkinghoff-Borg J., Lindorff-Larsen K. (2014). Combining Experiments and Simulations Using the Maximum Entropy Principle. PLoS Comput. Biol..

[cit50] Leung H. T. A., Bignucolo O., Aregger R., Dames S. A., Mazur A., Bernèche S., Grzesiek S. (2016). A Rigorous and Efficient Method To Reweight Very Large Conformational Ensembles Using Average Experimental Data and To Determine Their Relative Information Content. J. Chem. Theory Comput..

[cit51] Hermann M., Hub J. S. (2019). SAXS-Restrained Ensemble Simulations of Intrinsically Disordered Proteins with Commitment to the Principle of Maximum Entropy. J. Chem. Theory Comput..

[cit52] Bottaro S. , Bengtsen T. and Lindorff-Larsen K., in Structural Bioinformatics: Methods and Protocols, ed. Z. Gáspári, Springer US, New York, NY, 2020, pp. 219–240

[cit53] Yamamori Y., Tomii K. (2021). An ensemble reweighting method for combining the information of experiments and simulations. Chem. Phys. Lett..

[cit54] Hummer G., Köfinger J. (2015). Bayesian ensemble refinement by replica simulations and reweighting. J. Chem. Phys..

[cit55] Fisher C. K., Huang A., Stultz C. M. (2010). Modeling Intrinsically Disordered Proteins with Bayesian Statistics. J. Am. Chem. Soc..

[cit56] Sethi A., Anunciado D., Tian J., Vu D. M., Gnanakaran S. (2013). Deducing conformational variability of intrinsically disordered proteins from infrared spectroscopy with Bayesian statistics. Chem. Phys..

[cit57] Xiao X., Kallenbach N., Zhang Y. (2014). Peptide Conformation Analysis Using an Integrated Bayesian Approach. J. Chem. Theory Comput..

[cit58] Brookes D. H., Head-Gordon T. (2016). Experimental Inferential Structure Determination of Ensembles for Intrinsically Disordered Proteins. J. Am. Chem. Soc..

[cit59] Antonov L. D., Olsson S., Boomsma W., Hamelryck T. (2016). Bayesian inference of protein ensembles from SAXS data. Phys. Chem. Chem. Phys..

[cit60] Köfinger J., Stelzl L. S., Reuter K., Allande C., Reichel K., Hummer G. (2019). Efficient Ensemble Refinement by Reweighting. J. Chem. Theory Comput..

[cit61] Paissoni C., Jussupow A., Camilloni C. (2020). Determination of Protein Structural Ensembles by Hybrid-Resolution SAXS Restrained Molecular Dynamics. J. Chem. Theory Comput..

[cit62] Raddi R. M., Ge Y., Voelz V. A. (2023). BICePs v2.0: Software for Ensemble Reweighting Using Bayesian Inference of Conformational Populations. J. Chem. Inf. Model..

[cit63] Latham A. P., Zhang B. (2020). Maximum Entropy Optimized Force Field for Intrinsically Disordered Proteins. J. Chem. Theory Comput..

[cit64] Bertini I., Giachetti A., Luchinat C., Parigi G., Petoukhov M. V., Pierattelli R., Ravera E., Svergun D. I. (2010). Conformational Space of Flexible Biological Macromolecules from Average Data. J. Am. Chem. Soc..

[cit65] Bertini I., Ferella L., Luchinat C., Parigi G., Petoukhov M. V., Ravera E., Rosato A., Svergun D. I. (2012). MaxOcc: a web portal for maximum occurrence analysis. J. Biomol. NMR.

[cit66] Medeiros Selegato D., Bracco C., Giannelli C., Parigi G., Luchinat C., Sgheri L., Ravera E. (2021). Comparison of Different Reweighting Approaches for the Calculation of Conformational Variability of Macromolecules from Molecular Simulations. ChemPhysChem.

[cit67] Bonomi M., Heller G. T., Camilloni C., Vendruscolo M. (2017). Principles of protein structural ensemble determination. Curr. Opin. Struct. Biol..

[cit68] Orioli S. , Larsen A. H., Bottaro S. and Lindorff-Larsen K., Progress in Molecular Biology and Translational Science, in Computational Approaches for Understanding Dynamical Systems: Protein Folding and Assembly, ed. B. Strodel and B. Barz, Academic Press, 2020, pp. 123–176

[cit69] Kullback S., Leibler R. A. (1951). On Information and Sufficiency. Ann. Math. Stat..

[cit70] Boyd S. and Vandenberghe L., Convex Optimization, Cambridge University Press, Cambridge, 2004

[cit71] Smith L. J., Sutcliffe M. J., Redfield C., Dobson C. M. (1993). Structure of Hen Lysozyme in Solution. J. Mol. Biol..

[cit72] Shannon C. E. (1948). A mathematical theory of communication. Bell Syst. Tech. J..

[cit73] Thomas A. , An introduction to entropy, cross entropy and KL divergence in machine learning, 2019

[cit74] Jaynes E. T. (1957). Information Theory and Statistical Mechanics. Phys. Rev..

[cit75] Jaynes E. T. (1957). Information Theory and Statistical Mechanics. II. Phys. Rev..

[cit76] Cover T. M. and Thomas J. A., Chapter Introduction and Preview, John Wiley and Sons, Ltd, 1991, pp. 1–11

[cit77] Wittenberg M. (2010). An Introduction to Maximum Entropy and Minimum Cross-entropy Estimation Using Stata. Stata J..

[cit78] Golan A. , Judge G. and Miller D. J., Maximum Entropy Econometrics: Robust Estimation with Limited Data, Wiley, 1996, ch. 2

[cit79] Park J. C., Abusalah S. T. (1997). Maximum Entropy: A Special Case of Minimum Cross-entropy Applied to Nonlinear Estimation by an Artificial Neural Network. Complex Syst..

[cit80] Goodfellow I. , Bengio Y. and Courville A., Deep Learning, 2016, https://www.deeplearningbook.org

[cit81] Abbas A. E., Cadenbach A. H., Salimi E. (2017). A Kullback–Leibler View of Maximum Entropy and Maximum Log-Probability Methods. Entropy.

[cit82] Bishop C. M. , Pattern Recognition and Machine Learning (Information Science and Statistics), Springer-Verlag, Berlin, Heidelberg, 2006, ch. 10.1.2

[cit83] Chan A., Silva H., Lim S., Kozuno T., Mahmood A. R., White M. (2022). Greedification operators for policy optimization: investigating forward and reverse KL divergences. J. Mach. Learn. Res..

[cit84] Vaitl L., Nicoli K. A., Nakajima S., Kessel P. (2022). Gradients should stay on path: better estimators of the reverse- and forward KL divergence for normalizing flows. Mach. Learn.: Sci. Technol..

[cit85] Gu Y. , Dong L., Wei F. and Huang M., MiniLLM: Knowledge Distillation of Large Language Models, *arXiv*, 2024, preprint, arXiv:2306.08543 10.48550/arXiv.2306.08543

[cit86] Shen M. and Diamant N., On KL Divergence in Discrete Spaces, https://argmax.blog/posts/kl-discrete/, accessed on 2024-08-14, 2022

[cit87] Stöckelmaier J., Capraz T., Oostenbrink C. (2025). Umbrella Refinement of Ensembles–An Alternative View of Ensemble Optimization. Molecules.

[cit88] Kozak M., Lewandowska A., Ołdziej S., Rodziewicz-Motowidło S., Liwo A. (2010). Combination of SAXS and NMR Techniques as a Tool for the Determination of Peptide Structure in Solution. J. Phys. Chem. Lett..

[cit89] Mead L. R., Papanicolaou N. (1984). Maximum entropy in the problem of moments. J. Math. Phys..

[cit90] Alexander G. , Statistical Mechanics of Complex Systems, Lecture Notes 3-4, The University of Warwick, 2010

[cit91] Cesari A., Gil-Ley A., Bussi G. (2016). Combining Simulations and Solution Experiments as a Paradigm for RNA Force Field Refinement. J. Chem. Theory Comput..

[cit92] Bottaro S. , Bengtsen T. and Lindorff-Larsen K., *Integrating Molecular Simulation and Experimental Data: A Bayesian/Maximum Entropy reweighting approach*, bioRxiv, 2018, preprint 10.1101/457952 32006288

[cit93] Gull S. F., Daniell G. J. (1978). Image reconstruction from incomplete and noisy data. Nature.

[cit94] Groth M., Malicka J., Czaplewski C., Ołdziej S., Łankiewicz L., Wiczk W., Liwo A. (1999). Maximum entropy approach to the determination of solution conformation of flexible polypeptides by global conformational analysis and NMR spectroscopy – Application to DNS1-c-[d-A2bu2, Trp4,Leu5]- enkephalin and DNS1-c-[d-A2bu2, Trp4, d-Leu5]enkephalin. J. Biomol. NMR.

[cit95] Jaynes E. T. , in Maximum-Entropy and Bayesian Methods in Science and Engineering: Foundations, ed. G. J. Erickson and C. R. Smith, Springer, Netherlands, Dordrecht, 1988, pp. 25–29

[cit96] Skilling J. , in Maximum Entropy and Bayesian Methods: Cambridge, England, 1988, ed. J. Skilling, Springer, Netherlands, Dordrecht, 1989, pp. 45–52

